# Polarized release of brain microvascular endothelial cell- derived extracellular vesicles is functionally coupled to leukocyte transendothelial migration

**DOI:** 10.21203/rs.3.rs-10119900/v1

**Published:** 2026-07-09

**Authors:** Dylan Krajewski, Shujun Ge, Evan R. Jellison, Yi Wu, Kalpani N. Udeni Galpayage Dona, Allison M. Andrews, Servio H. Ramirez, James L. McGrath, Samuel J.W. Chan, Ji-yu Zhu, Guillermo C. Bazan, Adam J. Adler, Joel S. Pachter

**Affiliations:** UConn Health; UConn Health; UConn Health; UConn Health; University of Florida; University of Florida; University of Florida; University of Rochester; National University of Singapore; National University of Singapore; National University of Singapore; UConn Health; UConn Health

**Keywords:** Neuroinflammation, Blood-Brain Barrier, Transendothelial Migration, Extracellular Vesicles

## Abstract

**Background:**

Abundant evidence indicates extracellular vesicles (EVs) (exosomes and microvesicles) are mediators of intercellular communication. Previous reports from this group further showed that EVs from brain microvascular endothelial cells (BMEC) transferred the tight junction protein (TJ) claudin-5 (CLN-5) to leukocytes *in vitro* and during experimental autoimmune encephalomyelitis, leading us to hypothesize that such interaction might facilitate transendothelial migration (TEM) by a “zipper mechanism” whereby CLN-5 molecules on leukocyte-bound EVs temporarily replace those at interendothelial junctions. Such a mechanism is likely to be under strict spatiotemporal control. A corollary to this is that EVs are released from BMEC in a polar manner, such that only those released from the apical surface interact with leukocytes, while those from the basolateral surface bind adventitial targets.

**Methods:**

To assess polar EV release from BMEC and subsequent BMEC EV:leukocyte interactions during TEM using transwell assays, flow cytometry, nanoparticle tracking, super-resolution and live-time imaging were used.

**Results:**

It was shown that EV release was highly polarized, as EVs accumulated predominantly in the chamber facing their membrane of origin. Supportive of polarized exosome release, apical and basolateral membrane material appeared segregated into discrete multivesicular body populations. Consistent with a role in TEM, apical-derived BMEC EVs preferentially bound leukocytes in a manner dependent on leukocyte adhesion, with TEM suppressed when EV release was inhibited. Polarized EV release was maintained under physiological flow, wherein CLN-5^+^ EVs exhibited near exclusive release at the apical surface, possibly reflecting their predisposition toward interacting with circulating immune cells.

**Conclusion:**

BMEC EVs are released in a polarized manner and those derived from the apical surface focally interact with adherent leukocytes. That inhibition of EV release further suppressed TEM suggests that BMEC EV:leukocyte binding is functionally coupled to the TEM process.

## Introduction

Extracellular vesicles (EVs) are a heterogenous group of cell-derived, nano-sized, lipid-encapsulated particles found in various biofluids that utilize the bloodstream as a medium to communicate molecular information, e.g., proteins, nucleic acids and lipids, between adjacent or distant cells [1, 2]. Endothelial cells, by virtue of their lining the interior of blood vessels, are uniquely situated to release EVs that target cells on either side of the vascular border [3], as well as be targeted by EVs secreted from circulating or stromal cells [4].

Endothelial-derived EVs have been reported to interact with blood-borne leukocytes [5] in addition to vascular smooth muscle cells [6]. As these two cell types lie on opposite sides of the vessel wall and serve vastly different roles, this would suggest a means to release EVs in a vectorial manner is operative in endothelial cells. Consistent with such directional EV release is that some endothelia – particularly at the blood-brain barrier (BBB) – exhibit significant apical-to-basolateral polarity, corresponding to luminal- and abluminal-facing plasma membranes, respectively [7, 8]. Such polarity, sustained, in part, by inter-endothelial junctional complexes that restrict movements of phosphoinositides and proteins [9, 10], is reflected by apical and basolateral endothelial surfaces displaying different molecular topographies. Segregating membrane molecules in this way is a functional requirement, creating distinct signaling domains to ensure that interactions between endothelial cells and both cellular and non-cellular elements occur at the proper vascular interface [11, 12], along with prescribing directional secretion or transport of soluble factors [13] and regulating hemodynamics [14]. Because EVs fundamentally serve as extensions of their donor cell and reflect its molecular composition [15], it is conceivable that endothelial-derived EVs would convey information in a similarly discriminatory fashion to that of the endothelial cells from which they originated. For example, EVs originating from the apical surface facing the blood might be expected to carry signals that favor interaction with blood cells, while those arising from the basolateral surface facing the tissue may harbor molecules that preferentially impact adventitial or parenchymal cells. In-line with this reasoning are findings that TNF-α-stimulated, cultured vascular endothelial cells release ICAM-1^+^ EVs that promote monocyte transendothelial migration (TEM) [16], and blood-borne endothelial-derived EVs bearing both ICAM-1 and tissue factor promote monocyte aggregation [17]. Several reports have also documented that a variety of highly polarized epithelial and endothelial cells, when cultured atop dual chamber, permeable inserts, secrete heterogeneous populations of EVs having distinct transcriptomes and proteomes into the respective apical and basolateral chambers [18–21]. In particular, upon activation, basolateral EVs from cultured human aortic endothelial cells displayed greater changes in the EV secretome, exhibiting enrichment of pathways specific to atherosclerosis [21]. A similar dual chamber culture approach further showed that epithelial release of EVs is asymmetric, with EVs accumulating at the apical surface bearing proteins that originate from the apical membrane, and those at the basolateral surface reflecting the basolateral membrane protein composition [22].

It nevertheless remains to be established whether endothelial cells do, indeed, release EVs in a vectorial manner, whether this reflects endothelial membrane polarity, and what factors might impact this process. Since flow-generated wall shear stress is a major determinant of endothelial phenotype [23, 24], it could be a decisive factor in directional EV release. Also unclear is what role, if any, vectorial release of endothelial EVs plays in leukocyte:EV interactions. Namely, do leukocytes preferentially interact with EVs released from one endothelial surface or the other, and do chemokines, which drive leukocyte TEM [25, 26] affect EV release? Spatiotemporal control of where, when and what EVs are released from endothelial cells could significantly influence cell migration and prove critical in regulating inflammation, embryogenesis and cancer metastasis [27]. In fact, the appearance of endothelial-derived tight junction protein claudin-5 (CLN-5) on circulating leukocytes *in vivo* during neuroinflammation, along with the demonstration of CLN-5^+^ EV binding to leukocytes *in vitro*, has been cause for speculation that endothelial-derived EVs may facilitate leukocyte migration across the BBB [5, 28, 29]. If this is the case, brain microvascular endothelial cells (BMEC) might be expected to asymmetrically release CLN-5^+^ EVs from the apical surface, as this would best support juxtacrine communication between the vascular endothelium and leukocytes.

To address these outstanding issues, BMEC that comprise the BBB were cultured in dual chamber formats under both static and physiological flow conditions. Following differential labeling of apical vs basolateral membranes with fluorescent dyes, EVs from each chamber were analyzed to determine if they are released asymmetrically, and if EV release is related to shear stress, chemokine exposure and presence of leukocytes – all elements to which endothelial cells would be subject *in vivo* during inflammation. Results indicate that BMEC EV release is highly polarized. Additionally, transmigrating leukocytes preferentially bind EVs derived from the apical BMEC surface. Such binding appears to occur in a juxtacrine manner, at the interface of leukocyte:BMEC adhesion, enforcing local targeting of EV cargo.

## Methods

### Mice

Wildtype C57BL/6 mice were purchased from Charles River Laboratories, Inc. Transgenic C57BL/6J mice, expressing reporter eGFP fused to CLN-5 protein (CLN-5-eGFP mice), under direction of the endothelial Tie-2/Tek-1 promoter/enhancer [30], were obtained from Dr. Dritan Agalliu (Department of Pathology, Columbia University). All animal experimental procedures were performed in accordance with the Animal Care and Use Guidelines of UConn Health (Animal Welfare Assurance A3471–01) and approved under protocols 200853–0126 and 201564–1128.

### Cell culture

Two sources of mouse brain microvascular endothelial cells (BMEC) were used. bEND.3 cells, an immortalized line derived from a mouse capillary hemangioma [31] that exhibits polarity when cultured on transwell filters [32] as well as expresses several tight junction proteins and reduced paracellular permeability reflective of the BBB [33], were obtained from ATCC and expanded in high-glucose DMEM supplemented with 10% FBS, 2 mM L-Glutamine and 100 U/mL penicillin/streptomycin (all Gibco). Cells were grown at 5% CO_2_ and passaged using 0.25% Trypsin-EDTA (Gibco). For all experiments, bEND.3 cells were used prior to passage 15.

Primary mouse BMEC were obtained from male and female wildtype C57BL/6 or CLN-5-eGFP mice, aged 8–12 weeks, according to published protocols from this group [5, 34]. Cells were first expanded to confluence on a 35 mm tissue culture dish coated with 1 mL of 100 μg/mL murine collagen IV (Corning) in DMEM-F12 supplemented with 10% Horse serum, 10% FBS, 2 mM L-Glutamine, 100 U/mL penicillin/streptomycin (all Gibco) as well as 100 μg/mL heparin sulfate (Millipore Sigma) and 100 μg/mL endothelial cell growth supplement (Corning). They were then passaged only one time for experimentation using 0.05% trypsin-EDTA.

### Antibodies

Specific sources, clones and dilutions for all antibodies used are listed in **Additional file 1: Table S1**. Further technical details on specific use of antibodies are listed, below, under sections corresponding to individual experiments.

### Analysis of EV release under static conditions

**Figure 1:**
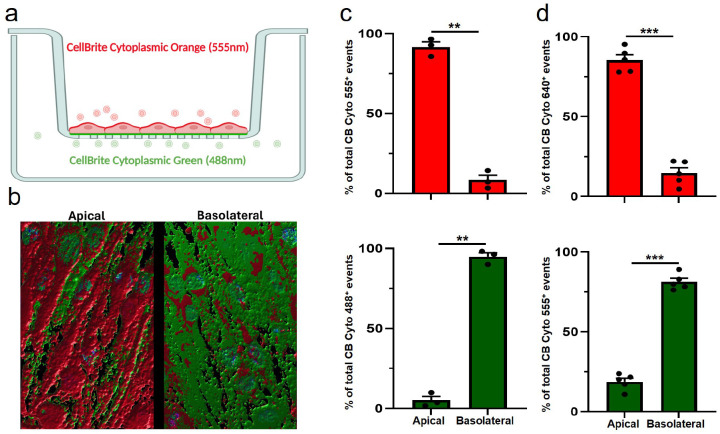
BMECs release total EVs in a polar manner under static conditions. **(a)** Labeling paradigm to measure distribution of EVs released by BMEC. CellBrite^â^ Cytoplasmic Orange/555 (red) and CellBrite^â^ Cytoplasmic Green/488 (green) were separately applied to the apical surface or basolateral surface, respectively, of BMEC cultured on dual-chamber inserts. Created in BioRender. Krajewski, D. (2026) https://BioRender.com/s8q1x94 **(b)** Isosurface-rendered image generated in Imaris^Ò^ from a confocal z-series of BMECs dual-labeled with CellBrite^â^ dyes. Red apical surface and green basolateral surface are depicted. Small areas of green shown on the apical surface, and red on the basolateral surface, are due to those regions of the membrane not being labeled intensely with CellBrite^â^ Cytoplasmic Orange or CellBrite Cytoplasmic Green, respectively, allowing the other dye to “peak through” from the opposite side. **(c)** Polar distribution of labeled total EVs isolated by ultracentrifugation at 100,000 × g from culture supernatant from either the apical or basolateral chamber of the insert, as determined by flow cytometry. Red indicates those EVs labeled with CellBrite^â^ Cytoplasmic Orange and originating from the apical surface; green indicates those EVs labeled with CellBrite^â^ Cytoplasmic Green dye and originating from the basolateral surface. **(d)** Polar distribution of labeled tetraspanin^+^ (FITC-CD63/CD81^+^) EVs isolated by ultracentrifugation at 100,000 × g from culture supernatant from either the apical or basolateral chamber of the insert, as determined by flow cytometry. Red indicates those tetraspanin^+^ EVs labeled with CellBrite^â^ Cytoplasmic Red and originating from the apical surface; green indicates those tetraspanin^+^ EVs labeled with CellBrite^â^ Cytoplasmic Orange dye and originating from the basolateral surface. The designations “Apical” and “Basolateral” underneath the bar graphs refer to the respective insert compartments from which the EVs were isolated. Bar graphs depict the mean ±SEM. All significance values were determined using paired, two-tailed t tests. **p <0.01; ***p<0.001.

For bEND.3 cells, cultures were trypsinized and seeded directly onto dual chamber, transwell filter inserts (1.0 μm pore size PET cell culture inserts [Falcon; item # 353103]) at confluence and allowed to attach for 48 h under normal growth conditions as described above. Apical vs basolateral surfaces were then differentially labeled using two separate CellBrite^→^ Cytoplasmic Membrane dyes (Biotium) (Fig. 1a). Briefly, staining solutions were made according to manufacturer instructions, and CellBrite^⊠^ Cytoplasmic Orange was applied to the apical surface while CellBrite^⊠^ Cytoplasmic Green was applied to the basolateral surface. After 20 min, free dye was washed out and cells were either fixed immediately for imaging or placed in growth media containing 10% exosome free FBS (Gibco) and 20 ng/mL TNF-α overnight. Supernatants were harvested from each chamber the next day for EV isolation and analysis. For tetraspanin stained samples, isolated EVs were costained with FITC-conjugated anti-CD63 and anti-CD81 for 30 min at 4 °C before analysis on the Amnis Imagestream X MKII (Cytek) flow cytometer. For primary BMEC, a similar protocol was used, but inserts were coated with 0.5 mL of 100 μg/mL murine collagen IV solution in 0.02 N HCl (Millipore Sigma) for 1 h and incubated in complete growth media overnight prior to cell seeding. Collagen IV from Millipore Sigma was used in this instance to maintain consistency with experiments performed under physiological flow conditions (see below).

### Analysis of EV release under physiological flow conditions

BE-DoubleFlow (BEOnChip; Zaragoza, Spain) cassettes were coated with 100 μg/mL murine Collagen IV solution (Millipore Sigma) for 1 h, washed, and subsequently coated with 100 μg/mL murine fibronectin in 0.1 M Tris-HCl, 0.15 M NaCl, pH 7.4 (Innovative Research). Of note, the Collagen IV used in this case was from a different provider than that described, above, for growth of primary BMEC in tissue culture dishes. This was because the shear stress due to physiological flow placed different adhesion demands on the cells, and the Collagen IV from Millipore Sigma better met these requirements while not being as satisfactory for supporting initial growth immediately following BMEC isolation. Collagen IV was used, as well as fibronectin, as the two together were proven necessary to facilitate strong cellular adhesion capable of withstanding the added stress of physiological flow. Primary murine BMEC were then seeded above confluence to compensate for the inability to thoroughly mix the cell suspension within the closed channel and cultured under static conditions for 48 h to allow for adherence. Cassettes were then connected to a peristaltic pump (Flocel Quad Pump) and subjected to a flow rate of 52 μL/min (0.23 dynes/cm^2^) overnight. The following day, the flow rate was gradually increased every hour until a flow rate of 630 μL/min (2.8 dynes/cm^2^), typical of post-capillary venules [35], was achieved. After 30 min of physiological flow, the peristaltic pump was disconnected to allow for cells to be dual-labeled using CellBrite^→^ Cytoplasmic dyes as described above. Alternatively, the apical surface of cells could be labeled under uninterrupted perfusion by delivering dye with a syringe pump (Fig. 7b, New Era Pump Systems NE-300), so as to avoid any unforeseen disruption in homeostatic function that may be associated with stopping flow along endothelial cells adapted to physiological hemodynamic forces [36]. After cell labeling, the pump was reconnected and initially set to the original low rate of 52 μL/min for 30 min to slowly reestablish flow, then gradually increasing by one pump speed setting (~ 100 μL/min on average) each hour before incubating cells in EV depleted media with 20 ng/mL TNF-α under physiological flow overnight. The following morning supernatants were taken for EV isolation and analysis on the Amnis Imagestream X MKII.

### EV isolation

EVs were isolated by a modified method of that previously described [5]. Cell culture supernatants were harvested separately from the apical and basolateral chambers of either filter inserts or flow cassettes, and then centrifuged at 8,000 × g for 30 min in an IEC Micromax 851 rotor to clear cellular debris and large vesicles such as apoptotic bodies. Supernatants were then transferred to Beckman Coulter thick-wall polycarbonate ultracentrifuge tubes (cat# 349622) and spun at 100,000 × g for 2 h using a TLA 100.3 fixed angle rotor in a Beckman Coulter TLA-100 benchtop ultracentrifuge to pellet total EVs. EV pellets were then resuspended in 50 μL of 0.1 μm-filtered PBS for downstream analysis. EVs were characterized by nanoparticle tracking analysis. Differential centrifugation was used in the case where different sized EV populations were resolved. Specifically, after clearing debris, cell culture supernatants were sequentially spun at 16,000 × g for 45 min to pellet large EVs, 60,000 × g for 1 h to pellet intermediate sized EVs, and 100,000 × g for 2 h to pellet small EVs.

### Nanoparticle tracking analysis

For concentration and size determination, EV isolates were brought up in 0.7 mLs of 0.1 μm-filtered PBS and run on the NS300 (Malvern Panalytical). Five × 1 min videos were recorded using a camera level of 12 and screen gain of 7, then analyzed using a detection threshold of 7 to exclude noise.

### Splenocyte isolation

Splenocytes were used as a source of leukocytes [37] for all experiments. Naïve wildtype C57BL/6 mice, or C57BL/6 female mice in which experimental autoimmune encephalomyelitis (EAE) was induced, were sacrificed by CO_2_ asphyxiation and spleens were immediately harvested and dispersed in complete culture media (RPMI 1640 with 10% heat inactivated FBS, 2 mM L-Glutamine, 100 U/mL penicillin/streptomycin, 10 mM HEPES pH 7.4, 1 mM sodium pyruvate, 50 μM 2-mercaptoethanol and non-essential amino acids) using the plunger of a 1 mL tuberculin syringe. Cell suspensions were then run through a 70 μm cell strainer (Fisherbrand) to remove residual connective tissue and spun down at 300 × g for 5 min. Pellets were resuspended in 5 mL red blood cell lysis buffer (eBioscience) and incubated with gentle agitation for 2 min before dilution in PBS. Cells were then pelleted again and resuspended and filtered a final time to yield a single cell suspension and cultured overnight in a 100 mm tissue culture dish (Falcon) prior to experimental use.

### Transendothelial migration (TEM) and EV:leukocyte binding experiments

bEND.3 cells were seeded onto dual chamber transwell inserts at confluence as described for previous experiments. However, to allow for leukocytes to more ably migrate across the filter; i.e., from the apical to basolateral chamber, a 3.0 μm pore size was used (Falcon; item # 353091). To prevent potential leaching of dye and artificial cell-to-cell transfer, the apical and basolateral surfaces of endothelial monolayers were labeled using CellBrite^→^ Fix dyes (Biotium), which, unlike CellBrite^⊠^ Cytoplasmic dyes, covalently link to membrane proteins after their lipophilic moiety embeds into the cell membrane. This ensures that any fluorescence signal detected on leukocytes is due to the exchange of actual membrane material from endothelial cell-to-leukocyte. After labeling endothelial cells, 2 × 10^6^ freshly isolated leukocytes from an EAE mouse were added to the apical chamber, and cocultures were maintained overnight in exosome-free media with 20 ng/mL TNF-α in both chambers as well as a cocktail of chemokines, including CCL2 (20 ng/mL), CXCL1 (10 ng/mL), CXCL13 (200 ng/mL) and CCL5 (4 ng/mL) (all from Biolegend), in the basolateral isolation and cells were stained with an immunophenotyping chamber to stimulate TEM of the different leukocyte subtypes (Fig. 3a). For leukocyte immunophenotyping, culture media was collected separately from the apical and basolateral chambers, and cells were pelleted by spinning at 300 × g for 5 min. Supernatants were saved for EV panel for 20 min at 4 °C. Finally, cell samples were analyzed on a FACSYMPHONY A5 SE flow cytometer (Becton Dickinson). Fluorescence compensation and analysis were performed in FlowJo version 10.10 (BD/Treestar) and gates were set based on unstained controls. EV isolates from the supernatant were run on the Amnis Imagestream X MKII to quantify the number of fluorescently tagged EVs remaining in suspension, and this number was used to calculate EV:leukocyte ratios for each respective membrane dye color.

To assess EV binding in the absence of direct endothelial and leukocyte contact, bEND.3 cells were grown and dual-labeled as described for transmigration experiments, then cultured overnight in the presence of chemokines and TNF-α with or without leukocytes from an EAE mouse in a cellular ratio equivalent to that used in the transmigration experiments (Fig. 5a,b). The following day, conditioned media from the apical chamber of each culture condition was taken, depleted of cells by centrifugation at 300 × g and applied to a proportional number of newly isolated EAE-leukocytes. Leukocytes and conditioned media were then incubated overnight on an uncoated 24 well plate with continuous agitation on an orbital shaker to prevent settling of cells and maximize the chance of EV interactions. After overnight incubation, leukocytes were fixed and stained as described above for flow cytometry analysis of EV binding.

### EV Flow cytometry

For EVs, samples were run on the Amnis Imagestream X MkII flow cytometer for 3 min per sample in high gain mode with a 60x objective to maximize signal intensity. Since accurate size determination is not possible on the Amnis for most EV populations, side scatter gates were created to collect anything near the lower limit of detection, but exclude large cellular debris. Gates were created based on unstained vesicles, and all fluorescence compensation and analysis were performed using single stained controls in Amnis Ideas software.

### Sub-cellular localization of multivesicular bodies (MVBs) generated from apical vs basolateral membranes

bEND.3 cells were seeded at confluence onto 1 μm pore size transwell inserts (Falcon) and cultured for 48 h prior to experimentation. To account for different rates of internalization, basolateral membranes were labeled first using CellBrite^→^ Cytoplasmic Green, then 1 h later apical membranes were labeled using CellBrite^⊠^ Cytoplasmic Orange. Cells were then incubated for an additional 2 h to allow for continued endocytosis of dye material and fixed for 5 min using 4% PFA. Fixed cells were then permeabilized with 0.1% saponin, blocked with 1% BSA and stained overnight at 4 °C with rabbit anti-Rab7 to tag MVBs, which were detected using anti-rabbit IgG-Alexa fluor 647. Finally, PET filters were excised from transwell inserts and mounted for microscopy using Mowiol (Millipore Sigma). After imaging on an LSM 880 confocal microscope (Zeiss), internalized puncta of each membrane dye were rendered in Imaris software version 10.2 (Bitplane) using the spot module, then filtered to puncta also containing Rab7 signal allowing us to highlight MVBs carrying membrane material from each cell membrane. Colocalization between MVBs containing either apical or basolateral membrane material was then defined as any two spots within 340 nm of each other.

### Surface rendering

bEND.3 cells were seeded at confluence on 1 μm pore size transwell inserts (Falcon) and cultured for 48 h prior to labeling apical vs basolateral surfaces using CellBrite^→^ Cytoplasmic Orange and CellBrite^⊠^ Cytoplasmic Green, respectively. Immediately after labeling, cells were washed with PBS, then fixed with 4% PFA for 5 min and DAPI stained. Filters were then excised and mounted with Mowiol for laser scanning confocal microscopy on an LSM 880 microscope. Using Imaris (Bitplane), fluorescent signal from each plasma membrane was rendered as an opaque surface at a threshold that excluded background signal.

### Live cell imaging of membrane colocalization during TEM

For these experiments, a μSiM dual chamber culture platform was modified to minimize basolateral chamber height to 75 μm, making it compatible with the high numerical aperture lenses needed for high resolution confocal microscopy. Ultrathin (~ 100 nm) silicon nitride membranes with a high density of 60 nm pores (0.6% porosity) and a lower density of 3 μm pores (1% porosity) were manufactured by SiMPore Inc. (West Henrietta, NY) and provided by the McGrath Laboratory. This dual-pore-size format served several purposes. The low density of 3 μm pores minimizes potential optical effects due to light scattering, while still allowing leukocyte migration into the basolateral chamber. The higher density of 60 nm pores supports unrestrained passage of chemokines and soluble factors across the membrane.

Modified μSiM frame and channel components were designed and purchased from Aline, Inc. and assembled as previously described [38]. μSiMs were mounted to a 4-well chamberglass (Cellvis) to support cell culture and microscopy applications. Assembled μSiMs were washed with sterile ultrapure water and apically coated with 100 μL of 0.1 mg/mL poly-L-ornithine solution (Millipore Sigma) for 30 min at 37 °C before seeding with 1.3 × 10^4^ bEND.3 cells. Cells were then incubated for 48 h to allow for cell attachment and barrier formation. After this period, bEND.3 cells were stained apically with COE-BY, a novel, water-soluble, amphipathic membrane dye obtained from Dr. Guillermo Bazan at the National University of Singapore. Briefly, a staining solution was made by diluting COE-BY to 2.5 μM in Hank’s balanced salt solution (HBSS). bEND.3 cells were washed twice with HBSS, and 100 μL of COE-BY solution was added to the apical chamber of the μSiM platform. The platform was then incubated for 15 min at 37 °C. Next, cells were washed two additional times with HBSS before proceeding with the experimental protocol.

For the TEM assay, leukocytes were isolated from a naïve mouse, activated overnight with 1 μg/mL anti-CD3e and 1 μg/mL anti-CD28 and stained with CellBrite^→^ Fix 640. A total of 7.5 × 10^4^ labeled leukocytes were then suspended in 100 μL of phenol red-free bEND.3 media containing 20 ng/mL TNF-α and 5% heat-inactivated FBS and added to the apical chamber. To minimize optical interference from culture media components during inverted microscopy, culture media was then washed from the basolateral chamber and replaced with live cell imaging solution (Molecular Probes) containing 20 ng/mL TNF-α and a chemokine cocktail of 100 ng/mL CCL2, 100 ng/mL CXCL1, 100 ng/mL CXCL12 and 200 ng/mL CXCL13. As a negative control to ensure accurate colocalization thresholding, control samples were prepared by culturing bEND.3 cells and leukocytes under identical conditions to those for experimental samples, but only staining the splenocyte population with CellBrite^⊠^ Fix 640 and leaving the bEND.3 cells unstained. Still images were then acquired for colocalization analysis.

Five hours after leukocyte addition, when peak occurrence of TEM events was observed by phase contrast, cells were imaged on a Nikon Crest V3 spinning disk confocal microscope using a 60X oil immersion lens. To create time lapse videos, images were acquired with a 5 min interval over the course of 30–50 min and Z stacks were set to include the apical endothelial cell membrane and leukocyte interface, cross sectioning each cell type to monitor their interaction in coculture. After acquiring time-lapse images, non-adherent leukocytes were retrieved by gently triturating and removing the apical media from imaged μSiMs and spotting it on a fresh 4-well chamberglass for imaging. Z stacks of non-adherent leukocytes were then taken using identical parameters to those described for adherent leukocytes. ROIs were chosen randomly, but were acquired only if devoid of apparent BMEC debris. Post-acquisition colocalization analysis was performed in Imaris with thresholds set for each channel based on background signal within the acquisition frame.

### SIM imaging of BMEC-derived EVs on the leukocyte surface

bEND.3 cells were seeded at confluence onto dual chamber 3.0 μm pore size transwell inserts and cultured for 48 h prior to experimentation. BMEC were then labeled with COE-BY as described above, and 2 × 10^6^ leukocytes freshly isolated from an EAE mouse with a score of 2.5 were added to the apical chamber along with 20 ng/mL TNF-α. The basolateral chamber was supplemented with 20 ng/mL TNF-α and the same chemokine cocktail previously used for live cell imaging of TEM.

After 16 h of coculture, leukocytes were harvested from the apical and basolateral chambers and fixed with 4% paraformaldehyde. The recovered cells were then blocked with 1% BSA and stained with FITC conjugated anti-CD63 and anti-CD81 overnight at 4 °C, counterstained with 1 μg/mL DAPI for 5 min at room temperature, and spotted on CellVis 4-well chambered cover glass (cat#: C4–1.5H-N). They were then imaged on a Zeiss Elyra7 in lattice SIM mode at 63X and subjected to SIM^2^ analysis. Maximum intensity projection images were then analyzed and exported from Imaris.

### EV inhibition

To inhibit EV release, the widely used EV inhibitor GW4869 [39–41] (Millipore Sigma) was initially dissolved in DMSO as a stock solution. The effects of GW4869 on cell viability were assessed by incubating bEND.3 cells with a range of inhibitor concentrations for 16 h, and then performing viability analysis by MTS assay (Promega) according to manufacturer instructions. The viability of leukocytes was separately assessed by MTS assay after 16 h of treatment with either 5 μM GW4869 or a vehicle control. To determine the extent of inhibition of EV release, bEND.3 cells were treated with either 5 μM GW4869 or a vehicle control for 16 h in exosome depleted media with 20 ng/mL TNF-α. Conditioned media was then collected and EVs sedimented by ultracentrifugation. Isolated EVs were then resuspended in 0.1 μm filtered PBS and analyzed on the NS300 (Malvern Panalytical) to determine EV concentration. As only the binding of fluorescently labeled EVs to leukocytes can be monitored during TEM assays, the extent of inhibition of fluorescent EVs from bEND.3 cells that were apically labeled with CellBrite^→^ Cytoplasmic Orange was also assessed by an analogous experiment, where isolated EVs were costained with FITC-conjugated anti-CD63 and anti-CD81 for 30 min at 4 °C before analysis on the Amnis Imagestream flow cytometer.

To examine the effect of EV inhibition on both acquisition of BMEC-derived EVs by leukocytes and their subsequent capacity to undergo TEM, bEND.3 cells cultured on transwell inserts were first apically labeled with CellBrite^→^ Fix 640. Then, the following was added to the TEM assay: 20 ng/mL TNF-α and either 5 μM GW4869 or a vehicle control to both the apical and basolateral chambers; chemokine cocktail to the basolateral chamber; and anti-CD3/CD28 activated leukocytes to the apical chamber. As in the initial TEM experiments, BMEC and leukocyte cocultures were incubated overnight before leukocytes were harvested and stained with Live-or-Dye 330/410 (Biotium) and anti-CD45-BV605 before being analyzed by flow cytometry. Gates were set based on unstained and FMO controls in FlowJo and the percentage of live, CD45^+^ leukocytes that acquired CellBrite^⊠^ Fix 640 was calculated. Additionally, the percentage of total leukocytes that underwent TEM into the basolateral chamber was calculated using reported counts from both chambers over the course of a 3 min analysis.

### Statistical analysis

All statistical analyses and graphs were generated in Prism software version 10.2.3. Information on specific statistical tests used are indicated in the figure legends for each experiment.

## Results

### EVs are released in a polarized manner from BMEC under static conditions and reflect membrane polarity

Initial experiments exploited transwell-style dual chamber filter inserts to investigate polar release of BMEC EVs under static conditions (Fig. 1), and employed the BMEC line, bEND.3 cells (hereafter, the generic term *BMEC* will be used throughout the **Results** and **Discussion** sections, while the term bEND.3 cells will be restricted to the **Methods**). Taking advantage of the apical-to-basolateral polarity (luminal-to-abluminal *in vivo*) present in BMEC [7, 8] and evident in bEND.3 cells [33, 42], apical vs basolateral BMEC surfaces were differentially labeled with lipophilic dyes, CellBrite^→^ Cytoplasmic Orange and CellBrite^⊠^ Cytoplasmic Green, respectively, in cells grown on transwell inserts (Fig. 1a). Due to diffusional barriers, among them tight and adherens junctions, restricting lateral mobility of membrane lipids and proteins [43], the two BMEC cell surfaces were ‘marked’ with the separate dyes (Fig. 1b), allowing for the membrane origin of EVs released into the culture supernatants to be determined.

Flow cytometry of total EVs retrieved from either the apical or basolateral chamber, showed EV release was highly polarized (Fig. 1c). Specifically, > 90% of total orange labeled EVs released were recovered from the apical chamber, while ~ 80% of total green labeled EVs released were obtained from the basolateral chamber. This trend remained when the retrieved EVs were additionally stained with FITC conjugated antibodies to the tetraspanin proteins and common EV markers CD63 and CD81 prior to flow cytometry (Fig. 1d). Doing so allowed tetraspanin^+^ events to be gated on before evaluating polarity, confirming that *bona fide* EVs are released in a polar manner. As orange EVs could only have originated from the apical BMEC surface, and green EVs from the basolateral surface, these results indicate the following asymmetry: EVs born of the apical membrane are near exclusively released at the apical surface, while EVs derived from the basolateral membrane are released at the basolateral surface. To rule out the possibility that the transwell insert, itself, may have physically obstructed the passage of EVs, giving a false impression of polarized release and/or accumulation of these vesicles in the dual chamber cultures, EV distribution across a bare filter was analyzed. Specifically, following the dual-labeling procedure described above, EV-containing conditioned media from each chamber was transferred to the respective side of an empty transwell insert (i.e., devoid of BMEC). After overnight incubation, EVs were isolated from each chamber as previously described, and analyzed by flow cytometry (**Additional file 2: Fig. S1**). Results show that, in the absence of BMEC, apically derived EVs placed in the apical chamber equilibrated between chambers, while basolaterally derived EVs added to the basolateral chamber failed to cross the filter. This disparity arguably stemmed from the lack of a sufficient driving force for EVs placed in the basolateral chamber to flow upward through the solution and filter. It can thus be concluded that polarity of apically derived EVs is due to the barrier function of the endothelial cells, and not the permeable support alone.

#### Both small and large EVs are released from BMEC in a polarized manner

**Figure 2:**
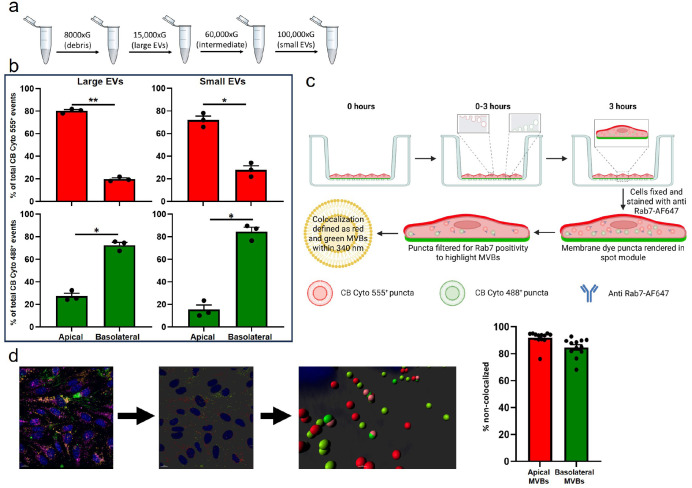
Small and large EVs exhibit polarized release from BMECs. Cells were labeled as in Fig. 1 **(a)** Differential ultracentrifugation protocol to resolve large and small EVs. **(b)** Polar distribution of labeled large and small EVs isolated separately from culture supernatant from the apical or basolateral chamber of the insert, as determined by flow cytometry. Red indicates those EVs labeled with CellBrite^â^ Cytoplasmic Orange/555 and originating from the apical surface; green indicates those EVs labeled with CellBrite^â^ Cytoplasmic Green/488 dye and originating from the basolateral surface. The designations “Apical” and “Basolateral” underneath the bar graphs refer to the respective insert chambers from which the EVs were isolated. **(c)** Labeling paradigm for resolving apical- and basolateral-derived late endosomes/MVBs. Red and green EVs < 340 nm apart from each other were defined as “colocalized”; red and green EVs > 340 nm apart from each other were designated as “non-colocalized.” Created in BioRender. Krajewski, D. (2026) https://BioRender.com/wqpw31r **(d)** Quantitative immunofluorescence of Rab7^+^ late endosomes/MVBs. Sequence of fluorescence images shows: *left*, raw fluorescence image; *middle*, image generated in the Spot Module (Imaris), depicting each late endosome/MVB as either a red or green sphere; *right*, enlarged Spot Module image. Bar graph indicates the percent of apically-labeled and basolaterally-labeled late endosomes/MVBs that are non-colocalized. Percentages are shown for both apical and basolateral MVBs, which differ from each other slightly due to differences in the abundance of each MVB population. Bar graphs depict the mean ±SEM. All significance values were determined using paired, two-tailed t tests. *p<0.05; **p <0.01.

The next studies determined if a specific size or subtype of EV undergoes polarized release (Fig. 2). To resolve the different populations, EVs were fractionated by differential centrifugation prior to flow cytometry (Fig. 2a). While several different subtypes of EVs are now recognized, exosomes and microvesicles constitute the major categories [44]. Exosomes are of smaller size, generally 50 nm – 100 nm in diameter, being derived from the endosomal pathway and released by exocytosis of multivesicular bodies (MVBs). By contrast, microvesicles (also referred to as ectosomes) typically tend to range from 100 nm in diameter – 1,000 nm in diameter, and arise by outward budding and pinching off of the plasma membrane. These size classifications are not definitive for EV subtype, however, as some exosomes can exhibit greater diameters than some microvesicles, and vice versa [45]. With specific regard to BMEC, the 20,000 × g spin yielded a mode size of ~ 170 nm and the 100,000 × g spin a mode size of ~ 125 nm, potentially reflecting both microvesicle and exosome populations (**Additional file 3: Fig. S2**). When referring to released or isolated EVs, the terms large and small EVs will be used from here on to designate those obtained at lower and higher g forces, respectively, and the terms microvesicles and exosomes reserved for discussing microscopic evaluation. EVs spanning both mode sizes appeared to be released in a similarly polarized fashion. Smaller and larger EVs originating from the apical membrane tended to be released at the apical surface. Likewise, smaller and larger EVs originating from the basolateral membrane equally favored release toward the basolateral side (Fig. 2b). Because microvesicles emerge directly from the cell surface, they would seem the most likely to undergo polarized release. However, since exosomes are generated by the endosomal pathway, their polarized release would appear more complex, necessitating that intracellular pools of MVBs derived from the apical vs basolateral membranes remain separate. To test this, confocal microscopy was used to perform a *two-step* colocalization analysis to determine if there was cytoplasmic mixing of MVBs containing endocytosed material from respective apical or basolateral membranes (Fig. 2c). The apical BMEC surface was labeled with CellBrite^→^ Cytoplasmic Orange, and the basolateral surface with CellBrite^⊠^ Cytoplasmic Green. After allowing for endocytosis, cells were fixed and MVBs identified by immunofluorescence using an antibody to Rab7, a marker for late endosomes/MVBs [46] – the source of “primordial exosomes.” In the first step, CellBrite^⊠^ Cytoplasmic Orange^+^ and CellBrite^⊠^ Cytoplasmic Green^+^ membrane dye puncta were separately rendered as discrete spots in Imaris and filtered to focus exclusively on puncta that were also Rab7^+^, representing MVBs derived from the apical and basolateral BMEC surfaces. In the second step, colocalization analysis was performed to quantify the percentage of pre-designated apical and basolateral MVBs that did not overlap with the other MVB population. Results from these experiments show that primordial exosomes derived from respective apical and basolateral membranes segregate within separate MVBs, as both membrane dyes colocalized with Rab7, but rarely with each other (Fig. 2d). Thus, despite exosomes from both apical and basolateral endothelial membranes congregating in close proximity within the cell’s interior, some sorting mechanism must exist to direct the release of exosomes at the corresponding cell surface.

#### During TEM leukocytes preferentially bind EVs released from the apical surface of BMEC

**Figure 3:**
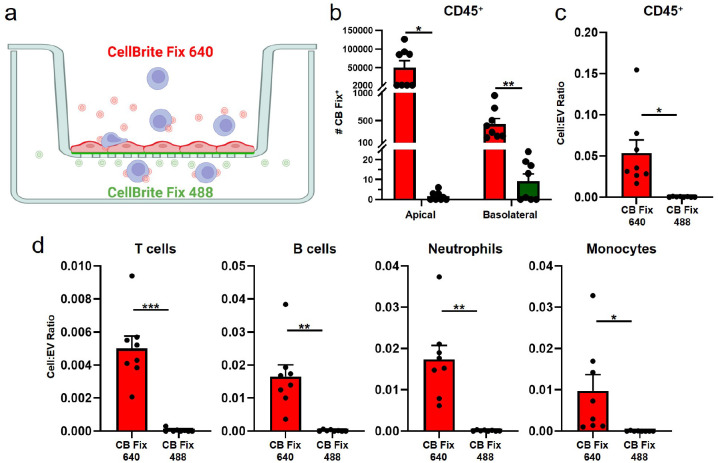
Leukocytes preferentially bind EVs released from the apical BMEC surface during TEM. **(a)** Labeling paradigm to measure binding of BMEC EVs to leukocytes. Apical and basolateral BMEC surfaces were differentially labeled as in Fig. 1, except that CellBrite^â^ Fix 640 (red) and CellBrite^â^ Fix 488 (green) dyes were used, respectively, to form covalent cross-links with plasma membrane proteins and avoid passive dye leakage. Unlabeled leukocytes were added to the apical chamber of the insert, and stimulated by a chemokine cocktail to undergo TEM. Created in BioRender. Krajewski, D. (2026) https://BioRender.com/9zho401 **(b)** Absolute # of red or green leukocytes in the two filter chambers following TEM, indicating those leukocytes that bound EVs (EV^+^ leukocytes), as determined by flow cytometry. **(c)** Normalized values of CD45^+^ EV^+^ leukocytes, showing red-labeled leukocytes/total red-labeled EVs or green-labeled leukocytes/total green-labeled EVs, as determined by flow cytometry. Values were obtained only for leukocytes in the basolateral chamber, as these represent the cells that underwent TEM. **(d)** EV^+^ leukocytes immunophenotyped by flow cytometry, and normalized as in (c). Bar graphs depict the mean ±SEM. All significance values were determined using paired, two-tailed t tests. *p<0.05; **p <0.01; ***p<0.001.

Since polarized release of BMEC EVs might afford unique opportunities for engagement with leukocytes, experiments were carried out to determine if EVs from the apical vs basolateral surface of BMEC preferentially associated with leukocytes during TEM (Fig. 3). To reflect in large measure the *in vivo* immune activation profile seen in an episode of neuroinflammation, leukocytes were obtained from spleens of mice that had been induced to develop the neuroinflammatory, demyelinating condition experimental autoimmune encephalomyelitis (EAE). Splenocytes were chosen as a leukocyte source due to high yield and representative immune cell profile, and will be referred to as leukocytes throughout. Immediately after splenic dissociation, cells were added to the apical chamber of transwell inserts containing confluent BMEC monolayers that were previously differentially labeled with fluorescent dyes on their apical and basolateral membranes, and a cocktail of chemokines was added to the basolateral chamber to encourage leukocyte TEM. In this case, however, CellBrite^→^ Fix 640 nm (red) and CellBrite^⊠^ Fix 488 nm (green) dyes were applied to the apical and basolateral surfaces, respectively, as they covalently bind to membrane proteins after embedding in the lipid bilayer (Fig. 3a). This binding property assured that any detectable transfer of fluorescent signal between endothelial cells and leukocytes would represent true exchange of proteinaceous membrane and not leakage of lipophilic dye. Because CellBrite^⊠^ Fix dyes label proteins, and do not distribute more ubiquitously among membrane lipids, the number of total labeled EVs released tended to be less than that seen with the CellBrite^⊠^ Cytoplasmic dyes. After 16 h of migration, leukocytes retrieved from both apical and basolateral chambers were found to have predominantly acquired CellBrite^⊠^ Fix 640 nm dye, while significantly fewer leukocytes from the basolateral chamber and virtually no leukocytes from the apical chamber picked up CellBrite^⊠^ Fix 488 nm dye (Fig. 3b; representative scatter plots shown in **Additional file 4: Fig. S3**). Additionally, there was a double-positive population of leukocytes that acquired both dyes, most likely reflecting these cells picked up both apically and basolaterally derived EVs. This group constituted a greater proportion of total CD45^+^ leukocytes than those that only bound basolaterally derived (CellBrite^⊠^ Fix 488) EVs. These results suggest that leukocytes greatly favor binding EVs released from the apical BMEC surface. However, to eliminate any differences in labeling efficiency of the two surfaces and, hence, unequal numbers of labeled EVs released into the apical vs basolateral chamber as possibly contributing to the observed disparity in leukocyte-EV binding, data were normalized as red-labeled leukocytes/total red-labeled EVs or green-labeled leukocytes/total green-labeled EVs (Fig. 3c). Data normalized in this way still revealed leukocytes demonstrate a preference for binding apically derived EVs.

Leukocytes from both chambers were also immunophenotyped (**Additional file 5: Fig. S4**) to determine if a particular leukocyte subtype showed any preference for acquiring BMEC EVs (Fig. 3d; **Additional file 4: Fig. S3**). Flow cytometry revealed monocytes, T cells, B cells, and neutrophils all demonstrated the ability to bind EVs released from BMEC, and a preference for the apically derived population of vesicles. However, while this capacity was universal among all immune cell types analyzed, neutrophils and monocytes bound the apically derived BMEC EVs with a much higher frequency, with 65–85% bearing the red fluorophore applied to the apical surface.

Studies were next carried out to determine if the cocktail of chemokines used to encourage leukocyte TEM had any direct effect on BMEC EV release or polarity. BMEC were again cultured on a transwell insert. Confluent monolayers were dual-labeled using CellBrite^→^ Cytoplasmic dyes, and cultured overnight with TNF-α ± chemokines in the absence of leukocytes. EVs were then isolated from apical and basolateral conditioned media by ultracentrifugation and analyzed by flow cytometry. Results show that exposure to a chemokine cocktail alone does not alter either the distribution or number of apically and basolaterally derived EVs (**Additional file 6: Fig. S5**).

### Apical BMEC membrane material acquired by leukocytes during TEM represents bona fide EVs.

**Figure 4:**
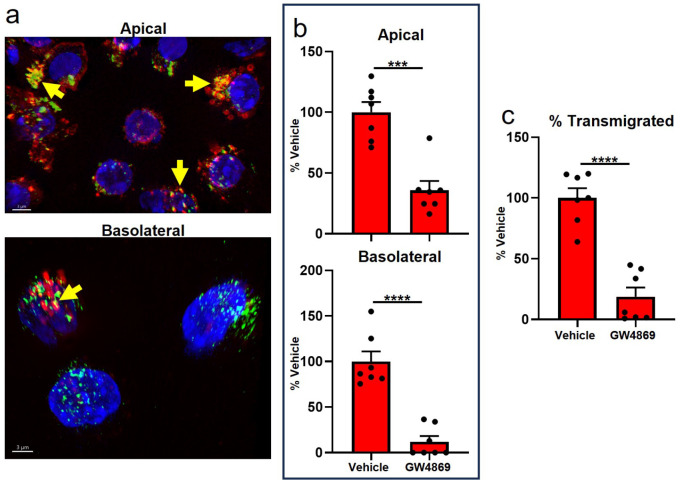
BMEC membrane signal acquired by leukocytes during TEM represents BMEC EV binding. **(a)** SIM microscopy showing COE-BY^+^ (red)/tetraspanin^+^ (green) (FITC-CD63/CD81^+^) BMEC EVs bound to leukocytes before (isolated from the apical chamber) and after (isolated from the basolateral chamber) undergoing transendothelial migration. Colocalized signal is shown in yellow. **(b)** Percentage of live, CD45^+^ leukocytes taken from the apical and basolateral chamber which are positive for BMEC derived CellBrite^â^ Fix 640 ± treatment with 5 μM GW4869 during TEM. Values shown are percentages relative to vehicle control. **(c)** Percentage of live, CD45^+^ leukocytes which have undergone TEM ± treatment with 5 μM GW4869. Values shown are percentages relative to vehicle control. Bar graphs depict the mean ±SEM. All significance values were determined using a two-tailed Student’s t test. ***p<0.001; ****p <0.0001.

Studies were next carried out to confirm that the fluorescent material bound to the migrating leukocytes indeed represented BMEC-derived EVs, and not merely randomly shed membrane debris (Fig. 4). First, the membrane dye signal acquired by leukocytes was analyzed by structured illumination microscopy (SIM) and SIM^2^ iterative processing. SIM was used to verify that membrane dye^+^ structures associated with leukocytes possess the tetraspanins CD63 and CD81, common markers for EVs. For this experiment, an analogous transmigration assay was performed as in Fig. 3, but a novel amphipathic membrane dye, COE-BY, was used in place of CellBrite^→^ Fix to apically label the endothelial monolayer. COE-BY is better suited for coculture imaging experiments as it is able to embed through both sides of the lipid bilayer and anchor in the cytoplasm [47, 48], thus preventing passive dye transfer while allowing for stronger and more uniform staining than protein cross-linking dyes. After the 16-h incubation period, leukocytes from the apical and basolateral chamber were fixed and stained with CD63/CD81 antibodies. SIM imaging revealed that numerous COE-BY^+^ puncta on the leukocyte surface were also tetraspanin^+^, consistent with their representing *bona fide* EVs (Fig. 4a). The superior resolution afforded by SIM^2^ analysis (60 nm, below the size of most EVs), additionally decreases the likelihood that the colocalized tetraspanin signal is present on neighboring leukocyte plasma membrane. Notably, there were also larger, submicron COE-BY^+^ structures bound to leukocytes that appeared spherical in shape, but did not express tetraspanins. These may represent greater-sized, BMEC-derived microvesicles, as this EV population is less associated with tetraspanins such as CD63 and CD81 [45].

Though apical-derived membrane from BMEC preferentially bound to leukocytes during TEM, and bore EV marker protein, the identity of the bound membrane could still be questioned. Also left unclear was the consequence of inhibiting EV release on leukocyte TEM, which might be expected to be curtailed if leukocyte binding of BMEC EVs were functionally coupled to diapedesis. Both issues were simultaneously addressed by first exposing BMEC/leukocyte cocultures to the widely used exosome inhibitor GW4869, and then employing flow cytometry to measure the impact on both EV-leukocyte binding and leukocyte TEM. As inhibition of EV formation and release can cause toxic effects dependent on cell type and density [39, 49], viability of BMEC and leukocytes was first independently assessed following exposure to a range of GW4869 concentrations (**Additional file 7: Fig. S6a,b**). For subsequent experiments a concentration of 5 μM GW4869 was selected, which was shown to significantly reduce BMEC EV release (**Additional file 7: Fig. S6c,d**) without causing toxicity. Compared to a vehicle control, the percentage of leukocytes that acquired BMEC-derived CellBrite^→^ Fix 640 was significantly reduced upon prior treatment with GW4869 (Fig. 4b). Additionally, the percentage of total leukocytes that transmigrated into the basolateral chamber was significantly reduced after GW4869 treatment (Fig. 4c). These data provide further support that acquisition of BMEC-derived membrane dye by leukocytes is representative of *bona fide* EV binding, not spurious attachment of membrane debris, and that this event could play a facilitative role in leukocyte TEM.

#### Binding of BMEC-derived EVs to leukocytes during TEM is context dependent

**Figure 5:**
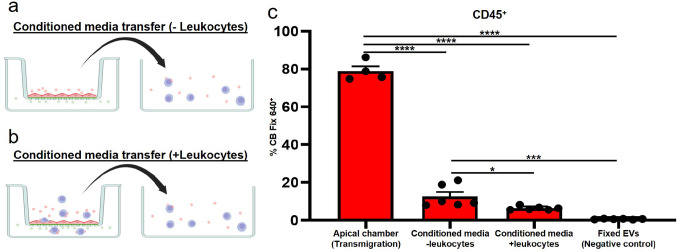
Leukocyte acquisition of BMEC EVs requires direct coculture. **(a-b)** Paradigms for evaluating leukocyte binding to EVs derived from BMEC that were cultured in the absence/− (top) or presence/+ (bottom) of leukocytes. As in Fig. 3a, BMEC were labeled with CellBrite^â^ Fix 640 to generate labeled EVs. Created in BioRender. Krajewski, D. (2026) https://BioRender.com/rwuh4gf; https://BioRender.com/4kz92kn **(c)** Percent of labeled CD45^+^ leukocytes resulting from the binding conditions highlighted in (a-b), as determined by flow cytometry. A negative control employing labeled EVs fixed with 4% paraformaldehyde was used as a negative control. Bar graphs depict the mean ±SEM. Significance values were determined by ordinary one-way ANOVA followed by Tukey’s multiple comparisons test. *p<0.05; ***p<0.001; ****p <0.0001.

Having established that leukocytes stimulated to migrate across BMEC in the TEM assay preferentially acquire apically-derived endothelial EVs (Fig. 3), the next series of experiments investigated if leukocytes must initially interact with endothelial cells in order to promote EV binding (Fig. 5). First, it was determined whether EVs released from BMEC exposed to inflammatory mediators, alone, could bind to leukocytes. To most closely mirror conditions of the TEM assay, BMEC were cultured on transwell inserts, labeled with CellBrite^→^ Fix dyes, and treated with chemokines and TNF-α, but not exposed to leukocytes. After 16 h, conditioned media from the apical chamber – which provides the greatest source of EVs capable of leukocyte binding during TEM – was harvested and cultured with leukocytes prepared from spleens of EAE mice exhibiting clinical scores equivalent to those in the earlier TEM studies (Fig. 5a). Uncentrifuged, conditioned media was used rather than purified EVs to control for any soluble factors that could potentially facilitate EV-leukocyte binding. And to most closely mirror the conditions of earlier TEM assays, the number of leukocytes added to BMEC-conditioned media kept the same ratio of leukocytes:media volume and leukocytes:BMEC present in the prior experiments. Moreover, to ensure that leukocytes had maximal opportunity to engage EVs, they were added to the EV-containing media in a microwell culture that was mixed on an orbital shaker for the entire incubation period. Results indicate that when exposed solely to conditioned media from the apical chamber of a transwell in which BMEC were exposed just to chemokine/cytokine, only **10%** of leukocytes acquired dye - presumably reflecting the limited binding of labeled EVs. By comparison, when a sample from the same leukocyte population was cultured directly with labeled BMEC in the TEM assay, **80%** of leukocytes became dye^+^. This suggests that the vast majority of BMEC EVs that bind to leukocytes during TEM do so as a result of leukocyte:BMEC interactions, and not simply because of cytokine stimulation. Nevertheless, a small population of BMEC EVs released into the apical chamber following chemokine/cytokine exposure, alone, appear to bind leukocytes by other than non-specific adsorption, as paraformaldehyde EV fixation abolished nearly all this activity (Fig. 5c).

To next clarify if prior exposure of leukocytes to BMEC was sufficient to generate EVs capable of binding to fresh leukocytes in the absence of BMEC, the identical format used for TEM was repeated. Leukocytes were added to BMEC previously labeled with CellBrite^→^ Fix dyes, and the configuration stimulated with chemokines and TNF-α. After 16 h, total media from the apical chamber was harvested and cleared of leukocytes by low-speed centrifugation, leaving free EVs in the supernatant. A fresh population of leukocytes was then added to the supernatant at the same ratio of leukocytes:BMEC used in the TEM assay and cultured overnight on an orbital shaker (Fig. 5b). Results indicate that when free BMEC-derived EVs generated by leukocyte:endothelial interactions were added to fresh leukocytes, they failed to achieve the extent of binding observed during the TEM assay (Fig. 5c). Thus, while leukocyte:endothelial interactions appear necessary to evoke the release of BMEC EVs capable of binding to leukocytes, conditioned media resulting from such interactions is not sufficient to recapitulate the TEM environment required for EV:leukocyte binding. The physical context in which leukocyte:endothelial interaction occurs during TEM might play an integral role in the EV binding process, perhaps requiring precise juxtacrine positioning of leukocyte and endothelial cell. *A priori*, separate endothelial EV populations generated by different means might also coordinate to impact leukocyte TEM at discrete steps.

**Figure 6:**
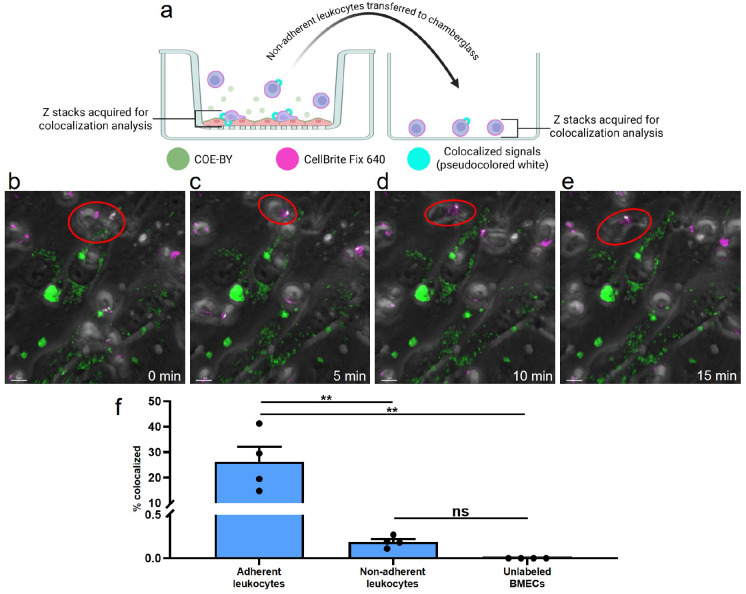
Leukocyte acquisition of BMEC EVs is linked to leukocyte:BMEC adhesion. (**a**) Experimental paradigm to image leukocyte (CellBrite^â^ Fix 640^+^) acquisition of BMEC EVs (COE-BY^+^) in live time on mSiM dual chamber casettes and compare the binding of BMEC EVs to BMEC-adherent or non-adherent leukocytes from during TEM assay (Created in BioRender). (**b**) Representative time-lapse images of an adherent leukocyte (designated by red circle) acquiring BMEC EVs and undergoing TEM with phase contrast imaging overlaid on fluorescent channels. Images are taken at 5 min intervals. To highlight areas of overlapping COE-BY and CellBrite^â^ Fix 640 signal, a colocalization channel was created and shown in white. (**c**) Quantification of colocalized signal among both adherent and non-adherent leukocytes, measured as depicted in (a). Colocalization was quantified as a percentage of CellBrite^â^ Fix 640^+^ voxels which were also COE-BY^+^ as thresholded based on the fluorescence intensity of background signal. A negative control group was also run where CellBrite^â^ Fix 640 labeled leukocytes were added to unstained BMECs. Bar graphs depict the mean ±SEM. Significance values were determined by ordinary one-way ANOVA followed by Tukey’s multiple comparisons test. **p <0.01, ns=*p*>0.05.

Since free BMEC EVs dispersed in the medium of the apical transwell chamber failed to achieve the degree of leukocyte binding observed during the original TEM assay – no matter if EVs were first elicited by prior leukocyte:BMEC interactions – it was reasoned that EV binding to leukocytes may be spatiotemporally linked to TEM. To test this, imaging experiments were performed in live-time with focus on the leukocyte:BMEC interface (Fig. 6a). To optimize our dual chamber model, BMEC were cultured on a modified microdevice featuring a silicon-nitride membrane (μSiM) cassette with a dual-pore, optically transparent silicon nitride nanomembrane, which, with a thickness of only 100 nm, provides superior optical properties compared with traditional PET permeable supports. COE-BY was again used to label BMEC, as its unique properties ideally lend themselves to coculture imaging. After BMEC labeling, a chemokine cocktail was introduced to the basolateral chamber, and leukocytes labeled with CellBrite^→^ Fix 640 added to the apical chamber, thus recapitulating our TEM model in a format conducive to high-magnification live cell imaging. The ~ 100× reduction in membrane thickness relative to conventional transwell filters eliminates the out-of-focus scatter that ordinarily precludes high-resolution imaging of the endothelial monolayer under oil-immersion objectives, making the leukocyte:BMEC apical interface directly accessible to spinning-disk confocal microscopy.

The μSiM platform allowed us to track leukocytes throughout the adhesion and migration stages of TEM by both phase contrast (**Additional file 8: Supplementary Video 1**) and fluorescence microscopy, wherein puncta consistent with the appearance of EVs, and carrying leukocyte-derived membrane dye could also be observed associated with nearby BMEC (**Additional file 9: Supplementary Video 2**). Notably, the acquisition of COE-BY^+^ EVs by leukocytes was observed after their adhesion to the BMEC monolayer (Fig. 6b–e, **Additional file 10: Supplementary Video 3**), consistent with physical leukocyte:BMEC interaction being the stimulus for BMEC EV release. Colocalization analysis showed that ~ 26% of CellBrite^→^ Fix 640^+^ voxels (corresponding to the leukocyte plasma membrane) were colocalized with COE-BY signal. Such extensive overlap of dyes is indicative of BMEC EV binding to the leukocyte membrane. To verify that the colocalization signal detected resulted from spatial overlap of the two membrane dyes, control samples were analyzed in which CellBrite^⊠^ Fix 640 labeled leukocytes were identically cocultured with unlabeled BMEC, and found to possess 0% colocalization using the same imaging and analysis parameters (Fig. 6f). Next, studies were performed to determine if the extent of BMEC EV binding, as measured by colocalization, was related to leukocyte:BMEC adhesion. After acquisition of fluorescent signal during the TEM assay, non-adherent leukocytes from the apical chamber of μSiM cassettes were taken and imaged on a separate chambered coverglass (Fig. 6a). Colocalization analysis revealed that the percentage of CellBrite^⊠^ Fix 640^+^ voxels from non-adherent leukocytes that colocalized with BMEC-derived COE-BY signal was drastically reduced compared to the colocalization observed in adherent leukocytes, showing a mean colocalization of only ~ 0.19% (Fig. 6f). This aligns with our previous findings from Fig. 5 and confirms that the interaction between BMEC EVs and leukocytes is context dependent, requiring that adhesion first occur between the two cell types in order for leukocytes to acquire BMEC EVs.

### Release of EVs from BMEC is highly polarized under physiological shear stress.

**Figure 7:**
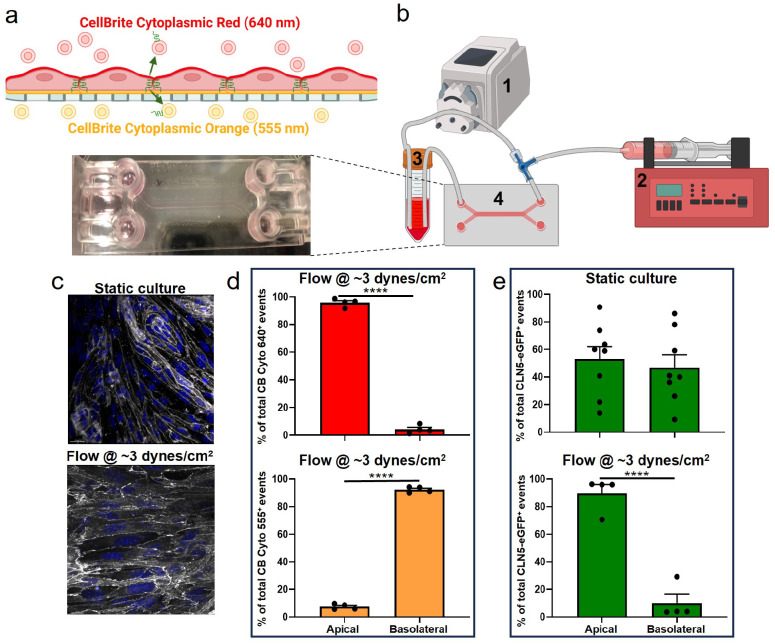
BMECs release total EVs and CLN-5^+^ EVs under physiological flow conditions. **(a)** Labeling paradigm showing unidirectional application of shear stress. Created in BioRender. Krajewski, D. (2026) https://BioRender.com/qfgie5t **(b)** Schematic of the components of the flow system: **1** peristaltic pump to apply shear stress to BMEC; **2** syringe pump to deliver membrane dye while maintaining shear stress; **3** bubble trap/media reservoir; **4** BEOnChip dual chamber cassette containing filter on which BMECs are grown (close-up photo shown under (a)). Created in BioRender. Krajewski, D. (2026) https://BioRender.com/zxw3yyw **(c)** Reorientation of BMECs parallel to the direction of flow/shear stress. **(d)** Polar distribution of labeled total EVs isolated by ultracentrifugation at 100,000 × g from culture supernatant from either the apical or basolateral compartment of the insert, as determined by flow cytometry. Red indicates those EVs labeled with CellBrite^â^ Cytoplasmic Red and originating from the apical surface; Orange indicates those EVs labeled with CellBrite^â^ Cytoplasmic Orange dye and originating from the basolateral surface. The designations “Apical” and “Basolateral” underneath the bar graphs refer to the respective insert compartments from which the EVs were isolated. (**e**) Polar distribution of eGFP-CLN-5^+^ total EVs. Bar graphs depict the mean ±SEM. All significance values were determined using paired, two-tailed t tests. ****p<0.0001.

As shear stress due to the frictional force of blood flow initiates endothelial signaling and significantly regulates function *in vivo* [50], it was next investigated if polarized EV release also occurred or was altered under physiological flow (Fig. 7). To additionally assess whether CLN-5^+^ EVs are released in a polar manner, as would be fitting for binding to circulating leukocytes and fostering TEM [5, 28, 29], primary BMEC derived from Tie-2-eGFP-CLN-5 mice [30] were used as the endothelial source. The lipophilic dyes CellBrite^→^ Cytoplasmic Red and CellBrite^⊠^ Cytoplasmic Orange were used for labeling apical and basolateral surfaces, respectively, analogous to the static studies with transwell inserts (Fig. 7a), but cells were cultured using a customized perfusion apparatus (Fig. 7b). As is a typical response of endothelial cells, the BMEC reoriented parallel to the application of flow/shear stress (Fig. 7c) [51]. When primary murine BMEC experienced shear stress similar to that found along postcapillary venules, the predominant site of leukocyte extravasation during inflammation [52], total EVs were released in polar fashion, as observed in static cultures (Fig. 7d). And CLN-5^+^ EVs, specifically, showed near exclusive release from the apical side of BMEC under flow, with ~ 80% being deposited in that direction (Fig. 7e). In marked contrast, when Tie-2-eGFP-CLN-5 BMEC were cultured under static conditions, CLN-5^+^ EVs were evenly distributed between apical and basolateral chambers. This indicates that while fluid shear stress is not a universal requirement for polar EV release, it may play a role in directing the vectorial release of specific EV subpopulations.

## Discussion

**Figure 8:**
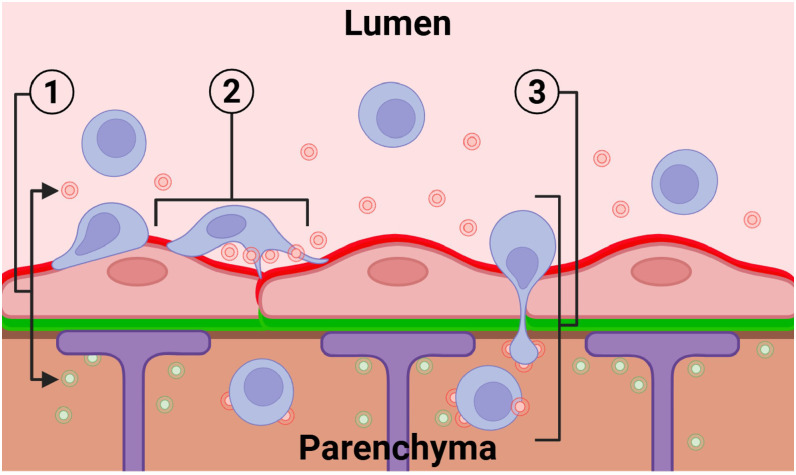
Schematic summarizes EV release from BMEC and interactions with leukocytes during TEM. An in vivo scenario is depicted showing orientation of vascular lumen and CNS parenchyma. Apical BMEC membrane and EVs are highlighted in red, basolateral BMEC membrane and EVs are highlighted in green. Illustrations of primary conclusions are numbered within figure. 1) EVs are released from BMEC in a polarized manner. 2) EVs released from the apical BMEC surface are trapped, and bind beneath the “umbrella” of an adherent leukocyte. 3) Leukocyte with bound EVs undergoes diapedesis. Created in BioRender. Krajewski, D. (2026) https://BioRender.com/8yut4yr

These studies employed unique differential labeling of apical vs basolateral membrane surfaces of cultured BMEC, to establish that EVs are released from a facsimile of the BBB in a highly polarized manner, with EVs originating from the apical membrane predominantly being released toward the apical side, and those arising from the basolateral membrane discharged toward the basolateral side. It was further shown that large- and small-sized EVs displayed polar release, and that EVs released from the apical BMEC surface, preferentially, bound to leukocytes undergoing TEM – particularly adherent cells poised to undergo diapedesis. Polarized EV release from BMEC was additionally observed under flow conditions and shear stress typically experienced by post-capillary venules, underscoring it occurs in a physiological hemodynamic environment that accompanies leukocyte TEM. A schematic summarizing these findings and how they relate to TEM is shown in Fig. 8.

Asymmetric release of EVs has previously been described in other settings. Using transwell cultures of polarized MDCK epithelial cells expressing transfected pan-neurotrophin receptor p75 or the vesicular stomatitis virus glycoprotein, proteins localized to the apical or basolateral surface, respectively, Colombo et al. (2020) reported that small EVs containing one or the other protein preferentially accumulated in the chamber facing the membrane from which each protein originated [22]. Wang et al. (2021), likewise, inferred polarized release of small EVs from human primary tubular epithelial cells stimulated with interferon and TNF-α, showing that the respective molecular cargoes (lipids, miRNA and proteins) of EVs collected from the apical or basolateral chamber of transwell filters substantially differed [53]. And, following extensive bioinformatic analysis, Raju et al. (2024) revealed small-sized EVs recovered separately from the apical and basolateral chambers of transwell cultures of human aortic endothelial cells (HAEC), as well as human umbilical vein endothelial cells, possessed distinct proteomes and siRNA transcriptomes [21].

The present work extends these findings, demonstrating that EVs of both small and large size are released from their surface of origin in highly polarized BMEC, and minimally – if at all – transit to the other side. Our ability to separately label the entirety of the respective endothelial surfaces, while retaining the capacity to generate small and large EVs, was key in distinguishing EV release from accumulation. In this regard, transit of EVs between the peripheral circulation and brain has been the subject of significant inquiry [54–56], raising the possibility that EVs released into one transwell chamber could ultimately accumulate in the other. Since the differential membrane labeling paradigm used here provided a means for both detecting EVs and identifying their membrane of origin, polarized release of EVs was confirmed. Importantly, such polarization was also verified under sustained flow and shear stress conditions approximating that found in post-capillary venules, underscoring that it can withstand physiological hemodynamic stresses, and can occur in the environment where leukocyte TEM takes place *in vivo*.

Quantitative, high-resolution image analysis also made clear that the cytoplasmic distributions of MVBs – the source of exosomes – originating from both apical and basolateral BMEC membranes were disperse but non-overlapping. The lack of congruence in the two MVB populations provides a cytological basis for our observation that both small and large EVs exhibited polarized release from BMEC. It was not surprising to detect polar release of large EVs, a population possibly reflecting microvesicles/ectosomes that bud from the BMEC plasma membrane, a boundary organized into well-defined and surface-restricted microdomains. The unequal partitioning of large EVs is also supported by a report that proteins involved in cell polarity of BMEC were confined to microvesicles [57]. Less expected, however, was the similar asymmetry shown by small EVs, as they’re more likely to represent exosomes derived from the endosomal pathway. Polarized release of exosomes from the surface of their origin would require that the two populations of MVBs bearing these EVs in elementary form remain distinct from each other in the cytoplasm, a situation consistent with our imaging data. That the vast majority of red membrane dye^+^/Rab7^+^ vesicles (MVBs derived from the apical surface) and green membrane dye^+^/Rab7^+^ vesicles (MVBs derived from the basolateral surface) were not coincident, suggests that pools of MVBs derived from opposite BMEC membranes, some of which engender primordial exosomes, are functionally sequestered and differentially tracked to the respective cell surfaces. This is in strong agreement with the finding of Colombo et al. (2020), who separately labeled apical and basolateral surfaces of polarized epithelial cells with two different size gold particles, and noted that very few MVBs, as viewed by transmission electron microscopy, possessed both markers [22].

Our study further established that all the major leukocyte subtypes can bind EVs when stimulated to undergo TEM *in vitro*. Moreover, leukocytes showed a decided preference for binding those EVs released at the apical BMEC surface, which reflects the luminal surface *in vivo*; i.e., that exposed to circulating blood elements. This is in accord with the report of Raju et al. (2024), which detailed *in silico*-generated interactomes reflecting preferred interactions *in vitro* between EVs obtained from the apical chamber of transwell HAEC cultures and isolated monocytes, and between EVs from the basolateral chamber and smooth muscle cells [21]. Such interactomes suggested that EVs released from the apical HAEC surface are destined to functionally engage blood elements *in vivo*, while EVs dispensed from the basolateral surface are fated to impact adventitial cells in the blood vessel wall. In keeping with the concept that cell surface origin determines EV tropism and effect, small EVs released from the basolateral surface of proximal tubular epithelial cells – the side exposed to the vascularized lamina propria *in vivo* – induced the highest secretion by peripheral blood mononuclear cells of cytokine IL-10 and chemokines MCP-1 and IL-8 [53]. Our results provide first-hand evidence for such a functional dichotomy of EV action, showing that leukocytes undergoing TEM exhibit a significant bias toward binding EVs released from the apical surface of BMEC. The unequivocal demonstration of leukocyte:EV binding serves to underscore that EVs have the potential to directly impact leukocyte behavior, and that such influence is not the result of soluble molecules, e.g., RNA and serum proteins [58, 59] that might adventitiously co-purify with EVs. Several additional observations further point to leukocytes that preferentially picked up apically labeled fluorescent BMEC membrane during TEM as representing a physiological binding of EVs at the blood:endothelial interface. *One*, treatment of BMEC with the exosome inhibitor GW4869 significantly reduced the percentage of transmigrated leukocytes bearing BMEC membrane signal. *Two*, as subcellular distributions of the two dyes were dispersed and non-overlapping, acquisition of fluorescent signal by transmigrated leukocytes is unlikely the result of immune cells having incidentally picked up cytoplasmic membrane fragments during transcellular migration, otherwise both dyes would have been expected to be more equally represented. *Three*, while transmigrated leukocytes could have picked up the few apically derived EVs that accumulated in the basolateral transwell chamber, the significantly diminished capacity of leukocytes to bind EVs in solution would disfavor this possibility. *Four*, the finding that treatment of EVs with the cross-linking fixative paraformaldehyde greatly reduced their binding to leukocytes, is consistent with protein structure on the EV surface being a determinant of this activity [60, 61]. *Five*, the significantly diminished capacity of isolated EVs to bind leukocytes in solution, compared to during TEM, reflects that context is a critical factor. At the incipient stages of TEM leukocytes are positioned over endothelial cells in way that might act as a canopy to prevent dilution of apically released EVs by the blood and maximize EV:leukocyte contact [5]. That EV release could be specifically directed towards sites of cellular union has been previously reported, as T cells were shown to release small EVs carrying distinct payloads into the T cell:APC immune synapse as compared to those released into the general extracellular space [62]. Induction of high-affinity leukocyte integrins by chemokines presented on the endothelial surface [63] may also support leukocyte:EV binding, as cognate counter ligands, e.g., ICAM-1, have been detected on endothelial EVs [64]. And in a tumor model, induction of high affinity LFA-1 on the surface of activated T cells has been shown to enable targeted binding of ICAM-1^+^PD-L1^+^ EVs to mediate T cell suppression [65]. ICAM-1^+^ endothelial EVs were further reported to stimulate monocyte TEM [16], possibly relating EV binding to leukocyte diapedesis.

While the release of BMEC EVs capable of binding to leukocytes appears dependent on leukocyte adhesion, leukocyte EVs may also be at play. EV-like puncta carrying leukocyte-derived membrane dye were observed associated with BMEC at sites of leukocyte adhesion, possibly representing leukocyte EVs that had been internalized or bound to the BMEC surface. This observation raises the prospect that EV exchange occurs at the leukocyte:BMEC interface in a type of juxtacrine, feed-forward/feed-back mechanism. In this case, EVs released by adherent leukocytes might stimulate the release of BMEC EVs, which, in turn, bind leukocytes prior to TEM.

Further evidence endorsing linkage of diapedesis to leukocyte binding of BMEC EVs was provided at two levels. Treatment of BMEC with the exosome inhibitor GW4869 significantly reduced the percentage of total leukocytes that had transmigrated. And, spinning disk confocal microscopy confirmed BMEC EV:leukocyte binding in live-time, supporting the hypothesis that this event is contextually restricted by leukocyte adhesion and coupled to the extravasation process. In particular, the preferential colocalization of BMEC-derived COE-BY^+^ structures with the membrane of adherent CellBrite Fix 640^+^ leukocytes on the apical BMEC surface, compared to non-adherent leukocytes in suspension, reinforced such a relationship. Furthermore, that at least some BMEC EVs remain bound to the surface of leukocytes, rather than being internalized, could portend a role in intercellular membrane interaction at the BBB, consistent with our hypothesis that such EVs may facilitate leukocyte TEM through tight junction complexes [5].

That polarized release of total EVs also occurred under flow validated the static transwell studies and may offer some insight into the relationship between EVs, junctional organization at the BBB, and leukocyte TEM. Surprisingly, EVs bearing the tight junction protein, CLN-5, did not exhibit polar release in the absence of flow but showed near exclusive discharge from the apical BMEC surface under conditions that recapitulated the wall shear stress experienced by post-capillary venules, the site of leukocyte extravasation *in vivo*. As both small and large EVs from BMEC have been shown to contain CLN-5 [5], this discrepancy might reflect some influence of shear stress on the signals that direct MVB:plasma membrane fusion [22] and/or repositioning of tight junctions along the inter-endothelial cleft. Regarding the latter possibility, in endothelial cells tight junctions can be found intermixed with adherence junctions [66], while in epithelial cells, tight junctions are concentrated at the most apical side of the junctional rim [9, 67]. *A priori*, flow-induced shear stress may relocate tight junctions to a more apical domain, thereby engendering the necessary molecular membrane asymmetry for polarized release of CLN-5^+^ EVs. The shear stress experienced by post-capillary venules may also impact the polarity of CLN-5^+^ EV release by reorienting tight junctions from perpendicular to parallel to the inter-endothelial cleft [8, 68]. Exclusive apical release of CLN-5^+^ EVs from BMEC would align with prior observations by this laboratory that circulating leukocytes obtain CLN-5 from endothelial cells as a possible means to navigate the BBB via a zipper mechanism employing transient homophilic CLN-5 binding between the two cell types [5, 29]. Occludin, another tight junction protein, and CD144 (VE-cadherin), an adherens junction protein, have also been detected on endothelial-derived EVs [69–71] during inflammatory conditions, reinforcing the prospect that EVs can serve as leukocyte:endothelial bridges mediated by junctional proteins spanning both cell types. Descriptions of endothelial EVs bearing other adhesion molecules prominent in leukocyte:endothelial adhesion, e.g. ICAM-1, VCAM-1 and CD31 [16, 72, 73], further supports an influential role for EVs in mediating the navigation of leukocytes through the vascular endothelium.

These collective results expand the potential realm of EVs as intercellular communicators [74–77], unveiling a cellular basis for asymmetrical crosstalk. By bearing features of the parent cell plasma membrane, EVs offer a means to extend the polar activities of BMEC beyond secreting or transporting molecules [7], to serving as physical interlocutors between endothelial and other cells specifically at the apical or basolateral surface.

## Conclusions

In summary, this study presents first-hand evidence that BMEC EV release is polarized under both flow and static conditions, wherein those EVs destined to bind leukocytes during TEM are discharged almost exclusively from the apical BMEC surface; i.e, the side that would be facing the blood *in vivo*. T cells, B cells, monocytes and neutrophils all exhibited binding of BMEC EVs. Binding of BMEC EVs was further observed to be largely restricted to adherent leukocytes, and pharmacological inhibition of EV release significantly reduced leukocyte TEM. Collectively, these results suggest that acquisition of BMEC EVs and leukocyte TEM are functionally coupled. Such a relationship is in line with previous work from this group reporting the binding of CLN-5^+^ BMEC EVs to leukocytes, a feature that could potentially impart leukocytes with the ability to interact with BMEC junctional proteins during paracellular migration.

## Supplementary Files

This is a list of supplementary files associated with this preprint. Click to download.

- AdditionalFile3.Fig.S2.jpg
- AdditionalFile6.Fig.S5.jpg
- AdditionalFile7.Fig.S6.jpg
- AdditionalFile5.Fig.S4.jpg
- AdditionalFile4.Fig.S3.jpg
- AdditionalFile1.TableS1.jpg
- AdditionalFile2.Fig.S1.jpg
- AdditionalFile10.SupplementaryVideo3.mp4
- AdditionalFile9.SupplementaryVideo2.mp4
- AdditionalFile8.SupplementaryVideo1.mp4
- Krajewskietal.2026SupplementaryfigurelegendsFBCNS.docx

## Data Availability

The data used and/or analyzed during the current study are available from the corresponding author upon reasonable request.
